# REFRACTORY BENIGN ESOPHAGEAL STRICTURES: A SYSTEMATIC REVIEW OF ENDOSCOPIC THERAPY

**DOI:** 10.1590/S0004-2803.24612026-027

**Published:** 2026-09-28

**Authors:** Aléxia Bezerra de Mendonça BELARMINO, José Jota Moura, Helena Aben-Athar PONTE, Franklin Freire Cartaxo ROLIM, Diego Bomfim SABOIA, Marcellus Henrique Loiola Ponte de SOUZA

**Affiliations:** 1 Universidade Federal do Ceará, Hospital Universitário Walter Cantídio, Fortaleza, CE, Brasil.; 2 Universidade de Fortaleza, Fortaleza, CE, Brasil.

**Keywords:** Esophageal stricture, surgical anastomosis, endoscopy, Estenose esofágica, anastomose cirúrgica, endoscopia

## Abstract

**Background:**

Refractory benign esophageal strictures (RBES) represent a significant clinical challenge due to their diverse etiologies and the variety of available therapeutic approaches. They affect approximately 30-40% of patients with recurrent strictures.

**Objective:**

To evaluate the efficacy of endoscopic therapies stratified by stricture etiology (anastomotic vs nonanastomotic).

**Methods:**

We conducted a systematic review in accordance with the PRISMA guidelines, including studies published between 2005 and 2025. Literature searches were performed in PubMed, ScienceDirect, and the CAPES Portal. Outcomes assessed included technical success, complication rates, and long-term clinical success. Study screening and data extraction were performed independently by two reviewers using Rayyan software.

**Results:**

Eighteen studies were included, comprising 208 individuals with anastomotic strictures (72.2% of studies) and 265 with nonanastomotic strictures (77.8% of studies). Therapeutic modalities included bougie dilation (7 studies; 38.9%), balloon dilation (7; 38.9%), triamcinolone injection (2; 11.1%), mitomycin C (3; 16.7%), stricturotomy (6; 33.3%), stent placement (10; 55.6%), and self-dilation (1; 5.6%). The overall technical success rate was 84.1%, whereas clinical success was 58.1%. Anastomotic strictures were associated with a worse prognosis, exhibiting a technical success rate of 89.4%, a complication rate of 33.2%, and a clinical success rate of 53.8%. Nonanastomotic strictures showed a technical success rate of 80.0%, a complication rate of 11.3%, and a clinical success rate of 61.1%. Four definitions of refractoriness were identified; the most common was the failure to achieve a diameter of 14 mm to 15 mm or greater after multiple dilations (9 studies) and repeated dilations without quantified criteria (5 studies).

**Conclusion:**

RBES demonstrate high technical success rates across therapeutic modalities; however, long-term clinical success remains limited and varies according to etiology. Anastomotic strictures are associated with a worse prognosis than nonanastomotic strictures, likely reflecting the complexity of postsurgical fibrotic remodeling. The marked heterogeneity in definitions of refractoriness across studies represents a critical limitation in the current literature.

## HIGHLIGHTS

• To systematically evaluate the effectiveness and safety of endoscopic therapies for refractory benign esophageal strictures.

• A systematic review of published studies assessing technical success, clinical outcomes, and complications of endoscopic treatment modalities.

• Endoscopic therapies demonstrated high technical success rates; however, long-term clinical success varied significantly according to stricture etiology.

• Stricture etiology plays a central role in treatment durability, highlighting the need for standardized definitions and well-designed prospective studies.

## RESUMO

**Contexto:**

Estenoses esofágicas benignas refratárias (RBES) representam desafio clínico significativo com múltiplas etiologias e modalidades terapêuticas. Afetam 30-40% dos pacientes com estenose recorrente. Objetivo: Avaliar eficácia de terapias endoscópicas estratificadas por etiologia (anastomótica vs não anastomótica).

**Métodos:**

Revisão sistemática conduzida a partir do guideline PRISMA, com estudos publicados entre 2005-2025, buscados nas plataformas PubMed, ScienceDirect e CAPES, com desfechos de sucesso técnico, complicações e sucesso clínico a longo prazo. Dois revisores independentes realizaram triagem e extração de dados utilizando o software Rayyan.

**Resultados:**

Foram analisados 18 estudos, nos quais 208 indivíduos apresentaram estenoses de etiologia anastomótica (72,2% dos estudos) e 265 de etiologia não anastomótica (77,8% dos estudos). Dentre as modalidades terapêuticas, foram relatadas dilatação com *bougie* (7 estudos, 38,9%), dilatação com balão (7 estudos, 38,9%), injeção de triancinolona (2 estudos, 11,1%), mitomicina C (3 estudos, 16,7%), estenotomia (6 estudos, 33,3%), colocação de *stents* (10 estudos, 55,6%), autodilatação (1 estudo, 5,6%). Taxa geral de sucesso técnico: 84,1%, porém, sucesso clínico: 58,1%. Estenoses anastomóticas apresentaram pior prognóstico, com sucesso técnico de 89,4%, complicações de 33,2%, sucesso clínico de 53,8%. Não anastomóticas demonstraram sucesso técnico de 80,0%, complicações de 11,3%, sucesso clínico de 61,1%. Quatro definições diferentes de refratariedade foram identificadas: incapacidade de atingir diâmetro ≥14-15 mm após múltiplas dilatações (9 estudos) e múltiplas dilatações sem critério quantificado (5 estudos).

**Conclusão:**

As estenoses avaliadas alcançam sucesso técnico elevado, mas sucesso clínico a longo prazo reduzido e dependente da etiologia. Estenoses anastomóticas apresentam pior prognóstico do que as não anastomóticas, o que reflete a complexidade cicatricial pós-cirúrgica. A heterogeneidade nas definições de refratariedade entre os estudos constitui uma lacuna crítica na literatura.

## INTRODUCTION

Esophageal strictures are defined as an abnormal narrowing of the esophageal lumen resulting from chronic inflammation. This inflammation leads to intramural fibrosis and scarring, resulting in a progressive reduction in the normal esophageal diameter-often to 13 mm or less-thereby impairing the passage of food^1^. The overall incidence of esophageal strictures is estimated at 1.1 per 10,000 person-years and increases progressively with age^2^. These strictures constitute a significant cause of dysphagia, considerably affecting oral intake capacity, nutritional status, and quality of life^3^.

Among nonanastomotic benign esophageal strictures, peptic strictures are the predominant type, accounting for approximately 70-80% of cases in adults. These result from chronic esophagitis induced by prolonged and poorly controlled gastroesophageal reflux disease. Conversely, caustic ingestion represents the leading cause of strictures in children and adolescents, particularly in developing countries, resulting in long-segment lesions that are difficult to manage endoscopically. Other causes include eosinophilic esophagitis, radiotherapy, esophageal trauma, systemic diseases (e.g., systemic sclerosis), graft-vs-host disease, and idiopathic strictures^2^ ^,^ ^4^.

Anastomotic strictures occur after esophagectomy, proximal gastrectomy, esophagogastric resections, and surgical repair of esophageal atresia. These represent a clinically relevant subgroup due to their potential for recurrence and the complexity of management^5^. Anastomotic strictures comprise one of the most frequent postoperative complications in children undergoing surgical repair of esophageal atresia. The reported incidence varies widely in the literature (32-80%), depending on the definition of stricture used and on multiple preoperative, intraoperative, and postoperative risk factors that influence anastomotic outcomes^6^. Recent studies have reported incidence rates ranging from 37.5-58.1%, whereas earlier reports described rates as high as 80%^7^.

The pathophysiology of strictures comprises multiple mechanisms, including the presence of long-gap esophageal atresia with consequent anastomotic tension-an important determinant of stricture formation and therapeutic response-as well as ischemia, anastomotic leakage, and surgical technical variables^8^. The initial anastomotic diameter is a critical predictor of the need for frequent dilations and the risk of refractory stricture; an initial diameter of 3 mm or less is associated with a greater than 20-fold increased risk of requiring stricture resection^7^.

Refractoriness represents a significant clinical challenge in the management of benign esophageal strictures, affecting approximately 30-40% of patients who experience recurrence after initial dilation^9^. Although studies have applied varying diagnostic criteria, the most widely accepted definition is that proposed by Kochman et al.^10^, which characterizes refractory strictures as those requiring more than five dilation sessions at 2-week intervals to achieve a diameter of 14 mm or greater in the absence of endoscopic evidence of inflammation. Failure to maintain an adequate esophageal lumen for 4 weeks after achieving a 14-mm diameter defines a recurrent stricture^11^. Independent risk factors for the development of refractory strictures include advanced patient age, stricture etiology (particularly anastomotic, caustic, and postradiation strictures), and the number and length of strictures^12^.

The treatment of refractory benign esophageal strictures (RBES) has evolved markedly over the past two decades, expanding from approaches based on repeated dilation to multiple adjuvant therapeutic strategies and minimally invasive procedures^8^. Endoscopic dilation remains the standard initial modality, with approximately 90% efficacy in simple strictures. Bougie techniques (Savary-Gilliard dilators) or balloon dilation demonstrate a technical success rate of 73.2%. Peptic strictures show better response rates, whereas postradiation, caustic, and surgical strictures typically require multiple dilation sessions^11^.

Self-expandable metallic stents (SEMS) are used both in refractory cases and as part of conservative management approaches. However, the overall clinical success rate is limited to 24.2% (14.1% for fully covered SEMS, 32.9% for biodegradable stents, and 27.1% for self-expandable plastic stents). Complications such as stent migration occur in 24.6% of cases, hyperplasia in 4.3%, stent impaction in 2.2%, and major complications in 17.7%^6^.

Regarding intralesional steroid injection therapy, triamcinolone reduces stricture recurrence by inhibiting fibroblastic proliferation. In post-esophagectomy strictures, triamcinolone decreases the number of required dilation sessions and is associated with lower recurrence rates^13^. In caustic strictures, it reduces the dilation index from 1.47 to 0.47, with concomitant improvement in dysphagia^14^. Esophageal self-dilation therapy (ESDT) has a learning technical success rate of 94% and decreases the number of endoscopic procedures from 17 to 1.5 (*P*<0.001), achieving a clinical success rate of 94% with a hematemesis risk of 6%^15^ ^,^ ^16^.

The incidence of therapeutic complications in endoscopic procedures for benign esophageal strictures is approximately 1% when all etiologies are considered, including perforation (0.1-2.6%), bleeding, and aspiration^17^. Despite recent advances, approaches to RBES remain controversial, particularly regarding etiology. This underscores the need for a systematic review focused on currently available endoscopic therapies. Accordingly, this review aims to synthesize evidence on therapeutic success rates and complications associated with endoscopic treatment of RBES, addressing the guiding question: “What is the efficacy of endoscopic treatments for refractory benign esophageal strictures?”

## METHODS

### Study design

this systematic review was conducted in accordance with the Preferred Reporting Items for Systematic Reviews and Meta-Analyses (PRISMA) guidelines. The review followed the PICO search strategy:

Population (P): adult and pediatric patients with RBES.

Intervention (I): endoscopic therapies, including balloon or bougie dilation, endoscopic electrosurgical incision therapy, intralesional steroid or mitomycin C injection, stent placement, or combinations of these modalities.

Comparison (C): anastomotic vs nonanastomotic etiology.

Outcomes (O): technical success rate, complications, and long-term clinical success.

Additionally, the definitions of refractoriness adopted in the included studies were evaluated.

### Eligibility criteria

randomized clinical trials (RCTs), nonrandomized clinical trials (NRCTs), observational studies (prospective or retrospective cohort studies and case-control studies), and case series including ≥3 patients were eligible for inclusion if they evaluated individuals with RBES undergoing endoscopic treatment and reported technical success rates, long-term clinical success, and treatment-related complications. Case reports, narrative or systematic reviews, qualitative studies, articles without full-text availability, discussion papers, letters to the editor, conference abstracts, expert opinions, books, book chapters, and studies that did not report outcomes relevant to this review were excluded.

### Search strategy

the search and analysis of eligible articles were conducted by two reviewers blinded to study outcomes between July and December 2025. Studies were identified through searches of the PubMed, ScienceDirect, and CAPES Portal databases using the descriptors “benign esophageal stricture”, “benign esophageal stenosis”, “endoscopic dilation”, “balloon dilation”, “bougie dilation”, “intralesional steroid”, “stent”, and “mitomycin C”. These terms were combined using the Boolean operators AND and OR, in both English and Portuguese. Eligible studies were published between 2005 and 2025.

### Data extraction and synthesis

title and abstract screening, as well as full-text evaluation, were conducted independently by two reviewers. Discrepancies were resolved by consensus or, when necessary, by consultation with a third reviewer. Eligible studies were extracted from each database, saved to a personal computer, and then exported to Rayyan software (Qatar Computing Research Institute) to assist with reviewer blinding, duplicate identification, and article screening.

Subsequently, the selected studies were read in full and stored in a new Microsoft Excel 2019 database (Redmond, WA). Extracted variables included year of publication, study design, country of origin, age range, number of patients with RBES, number of anastomotic and nonanastomotic strictures, stricture type and location, procedures conducted, technical success, complications, and long-term clinical success. The results were summarized descriptively using tables and presented narratively.

### Data analysis

data were analyzed using SPSS software, version 26 (Chicago, IL), and are presented as the total number of evaluated cases (*n*) and rates of technical success, complications, and long-term clinical success. As reported in the included studies, technical success of endoscopic dilation was defined as achieving the target diameter (generally ≥14-15 mm) for the stricture. For esophageal prostheses (esophageal stents), technical success was defined as appropriate device deployment. For intralesional corticosteroid injection (triamcinolone), technical success was considered complete injection into the planned quadrants of the stricture. For stricturotomy (endoscopic incision), technical success was defined as adequate completion of the planned procedures. For mitomycin C administration, technical success was defined as complete drug delivery to the stricture area.

Complications were defined as those reported by the studies, including perforation, fistula formation, bleeding, stent migration, tissue hyperplasia, significant postprocedural pain, and death. Long-term clinical success was defined as the resolution of dysphagia with tolerance of a normal diet. Rate calculations were conducted using the formula: (number of cases with technical success/complications/long-term success × 100) ÷ total number of RBES cases treated in the included studies. Results were presented by subgroup based on intervention type and study design.

### Study selection

the database search strategy identified 1129 potentially relevant articles. Of these, 121 duplicate records were removed, and 135 studies were screened for retrieval; 33 underwent full-text assessment. Fifteen studies were excluded due to nonendoscopic intervention (n=1), lack of reported success and complication rates (n=3), or inappropriate outcomes based on etiology (n=11). Ultimately, 18 studies were included in the systematic review (Figure 1).

**FIGURE 1:**
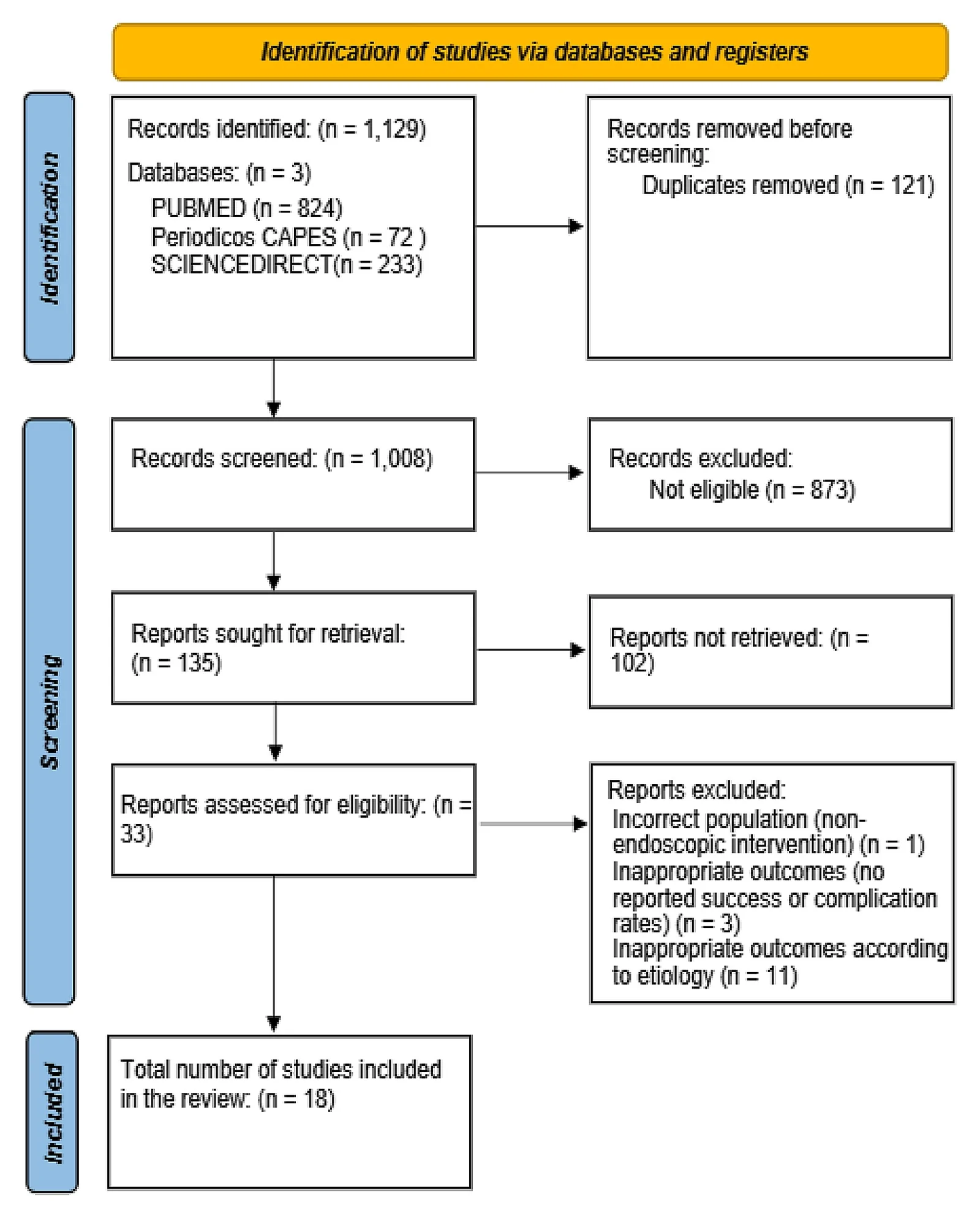
PRISMA Flowchart. *Consider, if feasible to do so, reporting the number of records identified from each database or register searched (rather than the total number across all databases/registers). *If automation tools were used, indicate how many records were excluded by a human and how many were excluded by automation tools.

## RESULTS

### Definitions

Refractoriness was classified into four main categories. The most frequently used definition (50.0%; 9 studies) was the inability to achieve a specific luminal diameter ≥14-15 mm after multiple dilation sessions. The second definition (11.1%; 2 studies) defined persistent clinical failure as ongoing dysphagia or inadequate oral intake despite multiple interventions. The third definition (27.8%; n=5 studies) described multiple dilations without specifying any quantifiable success criteria. The fourth definition was less frequently applied (11.1%; n=2 studies) and employed specific criteria related to feeding adequacy and demographic characteristics pertinent to the pediatric populations studied (Table 1).

**TABLE 1:** Definitions of refractoriness adopted in the included studies.

Authors	Group / General Definition	n (%)
Canakis et al.^12^, Ghobrial et al.^19^, Kahalekar et al.^20^, Kappelle et al.^21^, Liu et al.^22^, Nijhawan et al.^23^ , Repici et al.^24^, Wishahy et al.^25^, Wu et al.^26^	A - Failure to achieve diameter ≥14-15 mm after repeated dilations	9 (50.0)
Liu et al.^18^, Mitani et al.^27^	B - Persistent clinical failure (dysphagia/oral intake) after repeated dilations	2 (11.1)
Li et al.^28^, Madadi-Sanjani et al.^29^, Oh et al.^30^, Wang et al.^31^, Yano et al.^32^	C - Multiple dilations without clinical success	5 (27.8)
Awolaran et al.^33^, Manfredi et al.^34^	D - Specific criteria related to age and feeding adequacy	2 (11.1)

This systematic review evaluated 18 studies on the endoscopic management of RBES with anastomotic and nonanastomotic etiologies. The studies were conducted primarily in China (27.9%) and the United States (16.7%) and comprised prospective or retrospective cohort studies (44.4%), case series (38.9%), and clinical trials (16.7%). Regarding the study populations, 10 studies (55.6%) included adult patients, 6 (33.3%) pediatric patients, and 2 (11.1%) both children and adults. The number of patients per study ranged from 3 to 120, totaling 473 RBES cases; 208 were of anastomotic etiology (evaluated in 13 of 18 studies; 72.2%) and 265 were of nonanastomotic etiology (assessed in 14 of 18 studies; 77.8%), considering that nine studies included both etiologies in their analyses (Table 2).

**TABLE 2:** Methodological characteristics of the included studies.

Author	Year	Country	Study design	Age range	n	Etiology	
						Anastomotic	Nonanastomotic
Awolaran et al.	2020	United Kingdom	Case series	Children	3	1 (33.3)	2 (66.7)
Canakis et al.	2024	USA	Case series	Adults	4	1 (25.0)	3 (75.0)
Ghobrial et al.	2018	Egypt	RCT	Children	20	0 (0.0)	120 (100.0)
Kahalekar et al.	2017	India	Case series	Adults	1	0 (0.0)	11 (100.0)
Kappelle et al.	2019	Netherlands and Brazil	RCT	Adults	8	18 (100.0)	0 (0.0)
Li et al.	2024	China	Prospective cohort	Adults	1	4 (9.8)	37 (90.2)
Liu et al.	2015	China	Retrospective cohort	Children and adults	7	5 (71.4)	2 (28.6)
Liu et al.	2012	China	Retrospective cohort	Adults	24	24 (100.0)	0 (0.0)
Madadi-Sanjani et al.	2018	Germany	Case series	Children	11	9 (81.8)	2 (18.2)
Manfredi et al.	2018	USA	Retrospective cohort	Children	36	36 (100.0)	0 (0.0)
Mitani et al.	2024	Japan	Prospective cohort	Adults	54	33 (61.1)	21 (38.9)
Nijhawan, Udawat & Nagar	2015	India	Case series	Children and adults	11	0 (0.0)	11 (100.0)
Oh et al.	2010	USA	Prospective cohort	Adults	13	11 (84.6)	2 (15.4)
Repici et al.	2010	Netherlands and Italy	Prospective cohort	Adults	21	5 (23.8)	16 (76.2)
Wang et al.	2021	China	Case series	Children	14	0 (0.0)	14 (100.0)
Wishahy et al.	2019	Egypt	Case series	Children	17	0 (0.0)	17 (100.0)
Wu et al.	2019	China	Prospective cohort	Adults	50	50 (100.0)	0 (0.0)
Yano et al.	2017	Japan	NRCT	Adults	18	11 (61.1)	7 (38.9)

NRCT: nonrandomized clinical trial. Prosp: prospective. RCT: randomized clinical trial. Retro: retrospective.

Regarding stricture complexity, two studies (11.1%) described simple strictures, involving 21 cases (range, 3-18). Seven studies (38.9%) reported complex strictures, totaling 238 cases (range, 7-120), whereas 9 studies (50.0%) did not specify stricture complexity, totaling 214 cases (range, 3-54). Stricture location was reported in only 15 studies, accounting for 27.2% of the total cases. Among these, 47 cases were located in the proximal esophagus (range, 2-18), 38 in the midesophageal region (range, 3-20), and 44 in the distal esophagus (range, 2-14). The remaining 12 studies, comprising 72.7% of the total cases, did not provide specific information on stricture location, totaling 344 cases (range, 1-120) with unreported location (Table 3).

**TABLE 3:** Stricture characteristics of the included studies.

Variables	No. of Cases [Min-Max]
Type*	
Simple (2 studies)	21 [3-18]
Complex (7 studies)	238 [7-120]
Not specified (9 studies)	214 [3-54]
Location*	
Proximal (6 studies)	47 [2-18]
Midesophageal (4 studies)	38 [3-20]
Distal (5 studies)	44 [2-14]
Not reported (12 studies)	344 [1-120]

Among the therapeutic modalities, bougie dilation was conducted in seven studies (38.9%), balloon dilation in seven studies (38.9%), intralesional triamcinolone injection in two studies (11.2%), mitomycin C application in three studies (16.7%), stricturotomy (electrosurgical incision) in six studies (33.3%), and stent placement in 10 studies (55.6%). One study (5.6%) reported self-dilation as a therapeutic approach, and one study (5.6%) did not specify the intervention (Table 4).

**TABLE 4:** Types of procedures reported per study.

Authors	Procedures	n (%)
Ghobrial and Eskander, Kappelle et al., Liu et al., Oh et al., Wishahy et al., Yano et al.	Bougie dilation	7 (38.9%)
Awolaran et al., Canakis et al., Li et al., Liu et al., Madadi-Sanjani et al., Oh et al., Yano et al.	Balloon dilation	7 (38.9%)
Canakis et al.	Triamcinolone	2 (11.1%)
Canakis et al., Li et al., Liu et al., Manfredi et al., Wu et al., Yano et al.	Stricturotomy	6 (33.3%)
Ghobrial et al., Madadi-Sanjani et al., Wishahy et al.	Mitomycin C	3 (16.7%)
Awolaran, McGuirk & Arul, Canakis et al., Kahalekar et al., Kappelle et al., Liu et al., Oh et al., Repici et al., Wang et al., Wu et al.	Stent	10 (55.6%)
Li et al.	Self-dilation	1 (5.6%)
Mitani et al.	Others	1 (5.6%)

Thirteen studies (72.2%) included patients with anastomotic strictures, totaling 208 patients. The overall technical success rate for anastomotic strictures was 89.4% (186/208 cases), with rates ranging from 61.1-100% across studies. Complications were reported in 33.2% of cases (69/208), with inter-study variability ranging from 0% to 100%. The overall long-term clinical success rate was 53.8% (112/208 cases), with reported rates ranging from 0-100%. Seven studies reported clinical success rates of 50%-75%, whereas 5 studies reported rates below 30% (Table 5).

**TABLE 5:** Outcomes of anastomotic etiology strictures evaluated per study.

Author	Cases Anastom.	Technical Success	Complication	Long-term Success
		(%) (n=186)	(%) (n=69)	(%) (n=112)
Awolaran et al.	1	100.0	100.0	0.0
Canakis et al.	1	100.0	0.0	100.0
Kappelle et al.	18	100.0	44.4	27.8
Li et al.	4	100.0	0.0	0.0
Liu et al.	5	100.0	40.0	60.0
Liu et al.	24	100.0	50.0	75.0
Madadi-Sanjani et al.	9	66.7	0.0	66.7
Manfredi et al.	36	61.1	19.4	61.1
Mitani et al.	33	84.8	6.0	73.0
Oh et al.	11	100.0	72.7	18.2
Repici et al.	5	100.0	40.0	40.0
Wu et al.	50	100.0	50.0	52.0
Yano et al.	11	100.0	18.2	27.3
**Overall Rate**	208	**89.4**	**33.2**	**53.8**

Anastom: anastomotic.

Fourteen of the 18 studies (77.8%) provided specific data on patients with nonanastomotic strictures, totaling 265 cases. The overall technical success rate was 80.0% (n=212/265 cases), ranging from 0-100% across studies. Immediate complications occurred in 11.3% of cases (n=30/265). The overall long-term clinical success rate was 61.1% (n=162/265 cases), with inter-study variability ranging from 0% to 100%. Nine studies (64.3%) reported success rates greater than 50%, whereas 5 studies (35.7%) documented rates less than 50% (Table 6).

**TABLE 6:** Outcomes of nonanastomotic etiology strictures evaluated per study.

Author	Cases Nonanast.	Technical Success (%)	Complication (%)	Long-term Success (%)
		(n=212)	(n=30)	(n=162)
Awolaran et al.	2	100.0	100.0	0.0
Canakis et al.	3	100.0	0.0	33.3
Ghobrial et al.	120	60.8	0.0	60.8
Kahalekar et al.	11	100.0	36.4	54.5
Li et al.	37	100.0	0.0	56.8
Liu et al.	2	100.0	50.0	100.0
Madadi-Sanjani et al.	2	0.0	0.0	0.0
Mitani et al.	21	90.5	0.0	57.1
Nijhawan et al.	11	100.0	0.0	100.0
Oh et al.	2	50.0	50.0	50.0
Repici et al.	16	100.0	37.5	37.5
Wang et al.	14	100.0	57.1	92.9
Wishahy et al.	17	94.1	41.2	94.1
Yano et al.	7	100.0	14.3	0.0
**Overall Rate**	265	**80.0**	**11.3**	**61.1**

Nonanast: nonanastomotic.

## DISCUSSION

This systematic review analyzed 18 studies evaluating the endoscopic treatment of RBES, stratified by etiology and focused on technical success, complications, and long-term clinical success. The results demonstrate distinct outcome patterns between anastomotic and nonanastomotic strictures, with important implications for clinical practice and future research.

The present review identified four distinct categories of refractoriness definitions across the included studies. The most frequently used definition^12^ ^,^ ^19^ ^-^ ^26^ was the inability to achieve a diameter of 14-15 mm or greater after multiple dilation sessions, aligning with the internationally accepted classical criterion proposed by Kochman et al.^10^. This definition provides measurable objectivity and correlates with the capacity to swallow solid foods. The second definition^18^ ^,^ ^27^ prioritized clinical outcomes over luminal diameter measurement; Mitani et al.^27^ specifically defined stricture improvement as the point at which endoscopic dilation was no longer required. The third definition^28^ ^-^ ^32^ referred to multiple dilation sessions without explicit quantifiable criteria, thereby limiting reproducibility. The fourth definition^33^ ^,^ ^34^ employed criteria related to feeding adequacy and pediatric-specific demographic characteristics, reflecting the need for adapted definitions in pediatric populations. This heterogeneity in definitions partially explains the variability in reported success rates across studies.

This review revealed that patients with RBES treated endoscopically exhibit an overall technical success rate, with marked variation by etiology. These findings indicate that although most endoscopic procedures achieve the technical goal of stricture dilation, long-term durability is limited, suggesting distinct underlying pathophysiological mechanisms across etiologies. This interpretation is corroborated by the recent review by Chen et al.^8^, which indicates that dilation therapy is effective but that the lack of standardized parameters for dilation duration and pressure may result in inconsistent outcomes and increased complication rates.

Anastomotic strictures had higher technical success rates than nonanastomotic strictures. The observed difference of 9.4% may partially reflect the more uniform nature of anastomotic strictures, which result from a defined surgical event and often present with more predictable locations and characteristics. Baghdadi et al.^7^, in a retrospective review of 121 patients with esophageal atresia who underwent surgery between 2016 and 2019, found that the initial diameter of the esophageal anastomosis was predictive of the need for subsequent resection and repeated dilations. This indicates that lesion-specific and stricture-related characteristics significantly influence clinical outcomes.

Within the anastomotic subgroup, substantial heterogeneity was observed. Awolaran et al.^33^, Canakis et al.^12^, Kappelle et al.^21^, Li et al.^28^, Liu et al.^18^, Liu et al.^22^, Oh et al.^30^, Repici et al.^24^, Wu et al.^26^, and Yano et al.^32^ reported maximal technical success, suggesting that in selected clinical contexts and appropriately defined patient populations, endoscopic approaches can fully achieve technical objectives. In contrast, Manfredi et al.^34^, in a series of 36 children with postesophageal atresia anastomotic strictures treated with endoscopic electrosurgical incisional therapy (EIT), reported lower technical success rates despite a relatively low incidence of adverse events (5.3%; 7/133 EIT sessions). This dissociation indicates that more invasive procedures or treatment in highly complex pediatric populations may compromise technical success rates. Overall, the heterogeneity of these findings indicates that technical and pathophysiological variables related to the type of anastomotic stricture, as well as demographic factors such as patient age and postoperative interval, significantly influence initial technical success rates.

In the nonanastomotic context, Ghobrial and Eskander^19^, in an RCT involving 120 children with refractory caustic strictures comparing balloon dilation alone vs balloon dilation combined with MMC, reported a technical success rate of 60.8% in the MMC group, with a clinical response rate of 81.6% (49/60 patients free of dysphagia at 6 months). Conversely, Wishahy et al.^25^, in a cohort of 17 children with caustic strictures treated with endoscopic MMC infiltration, observed complete symptom resolution in 94% of cases (16/17), with a mean and median of only three additional dilation sessions required and improvement in the dysphagia score from 3 to 0. Li et al.^28^, in a study of 41 adults treated with esophageal self-dilation using a self-help inflatable balloon (SHIB), achieved maximal technical success; however, clinical success was observed in only half of the cases. When stratified by etiology, the endoscopic group (endoscopic submucosal dissection; n=30) achieved a clinical success rate of 63.3%, compared with 28.6% in the caustic group (n=7) and 0% in the surgical group (n=4). This demonstrates that etiology is an independent predictor of therapeutic response even when innovative technical modalities are employed.

Kahalekar et al.^20^, in a cohort of 11 adults with RBES (6 caustic, 3 peptic, and 2 postsclerotherapy strictures) treated with fully covered self-expandable metallic stents (FCSEMS), reported a technical success rate of 100% and an overall clinical success rate of 54.5% (6/11 cases). However, when stratified by etiology, 3 of 3 patients (100%) with peptic strictures responded, and 2 of 2 patients (100%) with postsclerotherapy strictures responded, whereas only 1 of 6 patients (16.7%) with caustic strictures achieved clinical success. These results highlight that caustic strictures have a markedly different prognosis, even when managed with identical therapeutic modalities.

The variability observed across studies reflects both methodological heterogeneity and the biological complexity of RBES. This pattern indicates that the specific etiology of the stricture, regardless of its classification as anastomotic or nonanastomotic, is a critical variable in determining technical success and may also vary with the duration of follow-up. Pediatric studies conducted by Manfredi et al.^34^, Ghobrial and Eskander^19^, Wishahy et al.^25^, Wang et al.^31^, Awolaran et al.^33^, and Madadi-Sanjani et al.^29^ reported lower technical success rates, possibly related to greater stricture complexity and reduced tolerance for prolonged procedures. Potential mechanisms include more aggressive pediatric etiology (e.g., caustic injury and anastomoses following esophageal atresia repair) and longer follow-up periods, which increase the likelihood of detecting recurrence.

Anastomotic strictures, often described as more complex and refractory, achieved slightly higher technical success rates. This may reflect the fact that anastomotic strictures are typically solitary and focal, allowing for targeted and effective dilation or incision. In contrast, nonanastomotic strictures-particularly caustic strictures-are frequently multifocal or diffuse, making complete treatment within a single session more challenging. Additionally, operators may have greater consolidated experience with anastomotic strictures, which are commonly encountered in high-volume centers, leading to more refined technical execution.

Complications represent a critical dimension often underestimated in discussions of treatment efficacy. The overall complication rate in this review was 21.8% (aggregated stratified data: anastomotic strictures, 33.2% [n=69/208]; nonanastomotic strictures, 11.3% [n=30/265]), exceeding rates reported for simple dilation in nonrefractory strictures. This 8-20% gap underscores the inherent risk of treating refractory strictures, reflecting chronic inflammation, extensive fibrosis, and the potential selection of cases with unfavorable anatomy. Anastomotic strictures exhibited a complication rate of 33.2%, nearly three times higher than that observed in nonanastomotic strictures (11.3%). This substantial difference indicates that prior surgical context, with associated anatomical alterations and deep fibrotic scarring, increases vulnerability to adverse events.

Kappelle et al.^21^, in an RCT involving 18 adults with post-esophagectomy anastomotic strictures randomized to 8 weeks of FCSEMS vs repeated bougie dilation, reported 8 adverse events among 9 patients treated with stents. Nonserious complications predominated, including chest pain in six patients, aspiration during stent removal in one patient, and stent-related reflux in one patient, compared with only minor complications in the dilation group. Mitani et al.^27^, in a cohort of 54 adults (33 anastomotic and 21 nonanastomotic) undergoing radial incision and cutting (RIC), reported an overall complication rate of 3.7% (2/54 patients), with 2 perforations in the anastomotic group managed conservatively. Notably, the nonanastomotic group exhibited 0% major complications, supporting the observation that RIC in nonanastomotic strictures, often characterized by different tissue fragility and underlying inflammatory profiles, may entail a distinct risk profile.

Oh et al.^30^, in a cohort of 13 US adults with anastomotic RBES treated with Polyflex (self-expandable plastic stents [SEPS]), found stent migration in 30% of cases (7/23 stents). Conversely, Wang et al.^31^, in 14 Chinese children with RBES treated with individually designed FCSEMS, documented stent migration or malposition in only 2 of 14 patients, with two additional cases of granulation tissue hyperplasia. These findings indicate that customized stent designs and refined techniques in pediatric populations may reduce migration rates compared with historical series using conventional devices. Stent migration remains a common complication of FCSEMS, with reported rates ranging from 7-75%, depending on specific stricture characteristics and stent deployment technique^35^. The overall rate of major complications associated with endoscopic dilation, including esophageal perforation, has been reported to range from 0.1-2.6% in a series of 474 dilation procedures^17^; this is lower than the rate observed in the present review. This outcome suggests that patients with RBES constitute a subgroup at increased risk compared with those with nonrefractory strictures.

A divergence between technical and long-term clinical success was observed, with a 27.1% gap. This discrepancy indicates that achieving the technical objective of stricture dilation or stent placement does not necessarily ensure sustained luminal patency or durable symptom relief. When stratified by etiology, this difference was even more pronounced in anastomotic strictures (gap of 35.6%) than in nonanastomotic strictures (gap of 18.9%). This finding reinforces that, despite adequate technical responses to endoscopic intervention, anastomotic strictures are disproportionately affected by recurrence or persistent dysphagia. The mechanisms underlying this difference may relate to the wound-healing process in the setting of deep fibrotic remodeling, amplified by prior surgery^1^.

Li et al.^28^ documented a recurrence rate of 48.8% (20/41) with SHIB use, despite reduced stricture length and prolonged postintervention intervals. Mitani et al.^27^ observed that despite only 9.5% achieving stricture improvement at 6 months in the nonanastomotic group treated with RIC, 57.7% achieved independence from balloon dilation over a 2-year follow-up period. This indicates that repeated RIC, combined with endoscopic dilation, may result in delayed success that is not captured in short-term assessments. Similarly, symptomatic recurrence despite maintained luminal patency was reported by Kappelle et al.^21^, who documented that 72% of evaluated patients (13/18) developed recurrent dysphagia during follow-up despite initial technical success, with a median time to recurrence of 36 days in the FCSEMS group vs 33 days in the bougie dilation group (*P*=0.576). These results indicate that the therapeutic modality has a limited influence on symptomatic recurrence rates in refractory anastomotic strictures.

Repici et al.^24^ and Yano et al.^32^, evaluating biodegradable stents, observed restenosis rates ranging from 45-66.7%, with only 16.7-45% of patients achieving sustained dysphagia-free status. These results indicate that even biodegradable stents, which avoid complications related to tissue embedment, fail to prevent fibrotic recurrence after stent degradation or removal. In contrast, Canakis et al.^12^ reported outcomes in patients with both anastomotic and nonanastomotic strictures who received a triamcinolone injection (40 mg/mL) into four quadrants as part of a triple-therapy approach, achieving complete technical success in both etiologies. However, one patient with a nonanastomotic stricture experienced only temporary resolution of solid-food dysphagia, with symptom recurrence occurring after 24 days.

The included studies employed mixed therapeutic strategies in their comparative analyses. Studies by Ghobrial and Eskander^19^, Kappelle et al.^21^, Liu et al.^22^, Oh et al.^30^, Wishahy et al.^25^, and Yano et al.^32^ used a Savary-Gilliard bougie or balloon dilation. Ghobrial and Eskander^19^, comparing balloon dilation alone (n=60) vs balloon dilation combined with MMC (n=120) in children, reported a 40% cure rate in the dilation-only group vs 81.6% in the MMC group, with a mean of 6.25 (SD, 1.74) dilation sessions required in the control group compared with 3.25 (SD, 2.78) in the MMC group (*P*<0.001). Kappelle et al.^21^, in nine adults randomized to repeated bougie dilation, reported a mean of 2.4 dilations over 12 months of follow-up, compared with 5.4 dilations in the stent group (*P*=0.159).

Awolaran et al.^33^, Canakis et al.^12^, Kahalekar et al.^20^, Kappelle et al.^21^, Liu et al.^18^, Liu et al.^22^, Oh et al.^30^, Repici et al.^24^, Wang et al.^31^, and Wu et al.^26^ included metallic stents. Kahalekar et al.^20^, in a cohort of 11 adults treated with FCSEMS, reported a 54.5% clinical success rate, with stent migration occurring in 36.3% of cases (4/11 stents). Kappelle et al.^21^, in a study of 9 adults randomized to FCSEMS placement for 8 weeks, reported 0% stent migration; however, clinical success did not differ significantly from that achieved with bougie dilation. Wang et al.^31^, in a cohort of 14 Chinese children treated with custom-designed stents adapted to individual patient characteristics, reported a 92.9% clinical success rate (13/14 patients free of dysphagia during follow-up), with only 14.3% experiencing stent migration or malposition. These results suggest that anatomy-tailored stent design, particularly in pediatric populations, substantially improves clinical outcomes. Liu et al.^18^, in a study of 24 adults, achieved a 75% clinical success rate (18/24 patients) at 12 months following treatment with a removable metallic stent.

Wu et al.^26^ compared 32 adults undergoing esophageal stent placement (ESP) with 18 treated by endoscopic incisional myotomy (EIM) for refractory anastomotic strictures. The ESP group achieved a significantly larger luminal diameter (19.9 [SD, 1.8] mm vs 11.0 [SD, 1.9] mm; *P*<0.001). However, improvement in dysphagia was greater in the EIM group (1.4 [SD, 0.5] vs 1.0 [SD, 0.0] points; *P*<0.001). At 12 months, 70% of ESP patients maintained luminal patency compared with 20% in the EIM group, highlighting a trade-off between superior immediate symptom relief with EIM and greater long-term durability with ESP.

Canakis et al.^12^, Li et al.^28^, Liu et al.^18^, Liu et al.^22^, Manfredi et al.^34^, and Wu et al.^26^ employed stricturotomy-based techniques. Manfredi et al.^34^, in a cohort of 36 children with refractory anastomotic strictures following esophageal atresia (EA) repair treated with EIT, reported a 61% success rate in the refractory group (22/36 cases), defined as requiring fewer than 7 dilations over 24 months. The median number of dilations required decreased from eight before EIT to two after EIT, with an adverse event rate of 5.3%. The authors further reported that an asymmetric scar contour was a better predictor of EIT success than the number of prior dilation sessions.

Mitani et al.^27^, comparing 21 nonanastomotic and 33 anastomotic patients treated with RIC, reported immediate improvement in dysphagia in 90.5% of the nonanastomotic group (19/21) vs 84.8% of the anastomotic group (28/33), with a median follow-up of 22.3 months. More importantly, 57.7% of nonanastomotic patients did not require additional endoscopic balloon dilation (EBD) at 2 years, despite only 9.5% revealing stricture improvement at 6 months, suggesting a durable benefit with a combined therapeutic strategy of repeated RIC plus EBD. Prior radiotherapy was identified as an independent risk factor for failure of stricture improvement in multivariate analysis (*P*=0.01).

Ghobrial and Eskander^19^, Madadi-Sanjani et al.^29^, and Wishahy et al.^25^ evaluated MMC. Ghobrial and Eskander^19^, in a study of 120 children, demonstrated an 81.6% cure rate (49/60) with MMC compared with 40% (24/60) with dilation alone (*P*<0.001), reducing the mean number of dilation sessions from 6.25 to 3.25 (*P*<0.001). Wishahy et al.^25^, in 17 children with refractory postcaustic strictures treated with endoscopic MMC infiltration, reported 94% complete resolution of dysphagia (16/17), with a mean of only 3.8 MMC-assisted dilations required and improvement in the dysphagia score from 3 to 0 (*P*<0.001). Conversely, Madadi-Sanjani et al.^29^ reported a success rate of only 55% (6/11) in children with postesophageal atresia or caustic strictures, with 45% (5/11) failing to respond and 2 patients requiring revision surgery. The authors indicated that the timing of MMC application (several months after injury in their series vs 12 weeks in other studies) may influence therapeutic response. This hypothesis is supported by the study of Machida et al.^36^, who, in a prospective series of 16 patients with refractory esophageal strictures following endoscopic submucosal dissection (ESD), documented a 62.5% success rate with topical MMC application after dilation, with a recurrence-free duration of up to 5 years among responders.

Awolaran et al.^33^, Repici et al.^24^, and Yano et al.^32^ used biodegradable stents. Repici et al.^24^, in a cohort of 21 adults treated with a biodegradable Ella stent, achieved a clinical success rate of 45% (9/20) over a median follow-up of 53 weeks, with a stent migration rate of 9.5% (2 cases occurring between weeks 4 and 7) and no major complications reported. Yano et al.^32^, in 21 adults treated with biodegradable stents for strictures following esophagectomy (n=11), ESD (n=6), or chemoradiotherapy (n=4), reported 66.7% dysphagia improvement (12/18) at 12 weeks, but only 16.7% maintained improvement at 24 weeks. Chen et al.^8^ observed that although stents may aid in remodeling scar tissue, they are associated with high rates of complications and migration, rendering their long-term effectiveness unsatisfactory.

Among studies reporting stricture location and complexity, a predominance of proximal, complex strictures was observed; however, most studies did not report these variables. This gap limits the ability to analyze correlations between stricture location, complexity, and therapeutic response. Studies examining pediatric EA^29^ ^,^ ^33^ ^,^ ^34^ frequently emphasize that cervical (proximal) anastomoses carry a higher risk of stricture formation and may differ in pathobiology from intrathoracic (distal) anastomoses. Similarly, the lack of morphological standardization hampers the comparison of therapeutic responses across stricture phenotypes, as more complex strictures tend to be associated with poorer outcomes.

This systematic review has some limitations that should be considered when interpreting the results. The heterogeneity of outcomes and the lack of therapeutic standardization observed across the included studies do not solely reflect the inherent biological uncertainty of esophageal strictures, but rather the methodological quality of the underlying literature. The predominance of observational studies over RCTs creates a significant evidence gap, precluding the formulation of definitive therapeutic recommendations or treatment algorithms. Although this review consolidates data from 473 published cases, it remains limited in its ability to provide high-confidence clinical guidance to inform practice. Critical advances in this field will depend on investment in high-impact methodological approaches, such as multicenter, prospective RCTs, rather than the continued accumulation of observational case series alone. Without such methodological advancement, the field of RBES is likely to remain characterized by technical fragmentation, nonstandardized intercenter variability, and the perpetuation of suboptimal therapies driven by local preference rather than rigorous evidence. Furthermore, there is an inherent risk of publication bias, in which studies reporting favorable outcomes are more likely to be published, while therapeutic failures may be underrepresented.

## CONCLUSION

This comprehensive systematic review synthesizes evidence on the efficacy of endoscopic therapies for RBES, with analyses stratified by anastomotic vs nonanastomotic etiology. The data reveal that although endoscopic therapies achieve high technical success rates, long-term clinical success remains modest and is strongly dependent on the underlying etiology. Anastomotic strictures demonstrated a markedly worse prognosis than nonanastomotic strictures, showing comparable technical success but lower long-term clinical success and higher complication rates. The heterogeneity in definitions of refractoriness across studies represents a critical gap in the literature. This lack of standardization limited robust pooling of outcomes and the generalizability of the findings. Well-designed RCTs are needed to better establish optimal therapeutic strategies for patients with refractory disease.

## Data Availability

data-available-upon-request
